# Computerized tomographic angiography for preoperative assessment of the superficial temporal artery for external carotid artery to internal carotid artery bypass: Case illustration

**DOI:** 10.1186/1757-1626-1-119

**Published:** 2008-08-21

**Authors:** Khashayar Farsad, Reyaad A Hayek, Alexander C Mamourian, Jonathan A Friedman

**Affiliations:** 1 Section of Neurosurgery, Dartmouth-Hitchcock Medical Center, Lebanon, NH, 03756, USA; 2 Section of Neuroradiology, Dartmouth-Hitchcock Medical Center, Lebanon, NH, 03756, USA; 3 Texas A&M Health Science Center College of Medicine 3201, Bryan, TX, 77802, USA; 4 Department of Radiology, Massachusetts General Hospital, Boston, MA, 02114, USA; 5 Department of Radiology, MSC10 5530, 1 University of New Mexico, Albuquerque, NM, 87131-0001, USA; 6 Department of Radiology, Hospital of the University of Pennsylvania, Philadelphia, PA, 19104, USA

## Abstract

**Introduction:**

While catheter angiography has traditionally been used to assess the caliber and course of the superficial temporal artery prior to its use as a conduit for external carotid artery to internal carotid artery (EC-IC) bypass, computed tomographic angiography (CTA) has become increasingly used in the diagnostic assessment of cerebral vasculature. We demonstrate the application of CTA for evaluation of the superficial temporal artery as a vascular conduit for EC-IC bypass.

**Case presentation:**

A female in the fourth decade of life presented with the chief complaint of headache. CTA of the Circle of Willis revealed an unruptured fusiform aneurysm of the M1 segment of the right middle cerebral artery (MCA). We performed CTA for the preoperative assessment of the STA for a putative EC-IC bypass procedure, and correlated this to conventional external carotid angiography. Reformatted CTA provided excellent surface visualization of the STA and its course in relationship to the cranial and zygomatic surfaces, and correlated well with findings on the conventional angiogram.

**Conclusion:**

CTA may eventually prove sufficient for use in assessing the STA in preparation for EC-IC bypass.

## Introduction

External carotid artery to internal carotid artery (EC-IC) bypass using the superficial temporal artery (STA) for cerebral revascularization is an important technique for a variety of complex cerebrovascular lesions. Invasive catheter angiography has traditionally been used to assess the caliber and course of the STA prior to its use as a conduit for EC-IC bypass. Computed tomographic angiography (CTA) has become increasingly used in the diagnostic assessment of cerebrovascular disease [1,2]. CTA has the theoretical advantages of avoiding the associated morbidity of catheter angiography while simultaneously decreasing time and cost. Conventional catheter angiography may be necessary in the evaluation of chronic ischemic disease to assess the distal cerebral vasculature and identify potential recipient vessels for bypass. However, catheter angiography could also potentially be avoided when bypass for intracranial aneurysm is planned. In this case illustration, we demonstrate application of CTA for evaluation of the superficial temporal artery as a vascular conduit for EC-IC bypass.

## Methods

A helical CT scan was performed on a 16-slice Light Speed Ultra (General Electric Medical Systems, Milwaukee, WI). Nonionic intravenous contrast media (Omnipaque 350 mg/dl) was injected into an antecubital vein at 5 cc/sec (total volume = 140 cc). After a standard delay of 15 seconds, helical CT scan of the brain commenced: Slice thickness 1.25 mm × 0.625 mm, pitch of 0.562:1, kV 140, mA 280, DFOV 16 cm. The standard CT angiogram dataset was then transferred to a Vitrea 2 v. 3.5 workstation (Plymouth, Minn,). Surface shaded display reconstruction directed for the evaluation of the External Carotid Artery branches was performed. Selective external carotid artery digital subtraction angiography was performed in a biplane angiography suite according to standard protocol. Both standard anteroposterior and lateral external carotid digital subtraction angiograms were obtained. The images were evaluated and compared independently by two neurosurgeons (J.A.F. and K.F.) and two neuroradiologists (A.C.M. and R.A.H.).

## Case presentation

**Figure 1:**
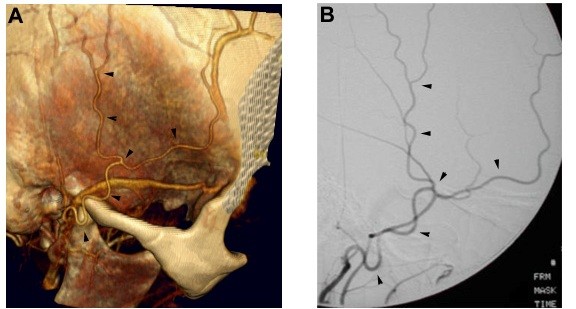
**Evaluation of the superficial temporal artery with CTA**. Comparison of the right superficial temporal artery (STA) as seen by CTA with surface-shaded display three-dimensional reformation (A), and by conventional invasive catheter angiography (B). Note the similarity of the STA as reproduced on the CTA compared with that seen on invasive catheter angiography. In addition, information with respect to relational anatomy with the skull and adjacent structures can be appreciated with the CTA.

A female in the fourth decade of life presented with the chief complaint of headache. A CT angiogram of the Circle of Willis revealed an unruptured fusiform aneurysm of the M1 segment of the right middle cerebral artery (MCA). Options for treatment included observation versus aneurysm trapping with extracranial to intracranial arterial bypass using the superficial temporal artery (STA) as the bypass conduit. We performed both CTA and conventional catheter angiography for the preoperative assessment of the STA for potential use in the proposed EC-IC bypass procedure. CTA provided excellent visualization of the STA and its course in relationship to the cranial and zygomatic surfaces (Fig 1A, arrowheads). The surface caliber and course of the STA as defined by CTA correlated well with the conventional catheter angiogram (Fig 1B, arrowheads). The patient ultimately decided on continued observation, and did not undergo the bypass procedure.

## Discussion

While the use of CTA to assess EC-IC bypass postoperatively has been described [3], we demonstrate how CTA may be used for the preoperative assessment of the STA for potential use in EC-IC bypass. Although use of preoperative CTA for anatomic evaluation of the external carotid anatomy has been described for head and neck surgery [4,5], to our knowledge, no report has yet been made demonstrating the utility of CTA for preoperative planning for EC-IC bypass. EC-IC bypass has remained one area where invasive catheter angiography has been thought necessary, specifically for evaluation of the STA vessel caliber as a bypass conduit. The patient described in this case illustration did not go on to surgery, therefore, no direct intraoperative comparison could be made between the findings at CTA and at DSA. To prove the utility of CTA for preoperative evaluation of the STA, intraoperative comparison of vessel caliber with DSA findings would be needed. If findings of vessel caliber at operation reliably compare with findings on CTA, the patient would be spared the additional risks and radiation exposure inherent to a DSA study, and the initial CTA to evaluate the aneurysm could also be used to evaluate potential external carotid artery conduits for bypass procedures. This would have important clinical implications, considering CTA is being used more and more for preoperative evaluation of cerebral aneurysms in general. The surface-rendering technique used in this study is likely to have some inherent inaccuracies with respect to vessel caliber when compared to volume-rendering; however, we feel this illustration provides a reasonable rationale for further study of the use of CTA in defining the course and caliber of the superficial temporal artery for EC-IC bypass.

## Conclusion

Catheter angiography remains useful for preoperative planning in many cases, such as planning for EC-IC bypass in chronic ischemic disease. Nevertheless, we feel that CTA may ultimately prove sufficient for use in assessing the STA in preparation for EC-IC bypass, given further supportive evidence with intra-operative comparison of vessel caliber.

## Competing interests

The authors declare that they have no competing interests.

## Authors' contributions

KF, JAF Manuscript formulation and preparation, figure arrangement, image interpretation, RAH, ACM Manuscript review, CT protocol, image acquisition and interpretation, 3D reconstructed image preparation. All authors read and approved the final manuscript.

## Consent

Written informed consent could not be obtained in this case since the manuscript was prepared retrospectively and the patient and next of kin were untraceable. We believe this case report contains a worthwhile clinical lesson which could not be as effectively made in any other way. We expect the next of kin not to object to the publication since the patient remains anonymous.
